# Age and gender specific cut-off points for body fat parameters among adults in Qatar

**DOI:** 10.1186/s12937-020-00569-1

**Published:** 2020-07-25

**Authors:** H. Bawadi, S. Hassan, A. Shanbeh Zadeh, H. Sarv, A. Kerkadi, Josep A. Tur, Z. Shi

**Affiliations:** 1 Department of Human Nutrition, College of Health Sciences, QU Health, Qatar University, Doha, 2713 Qatar; 2 Research Group on Community Nutrition & Oxidative Stress, University of the Balearic Islands & CIBEROBN, 07122 Palma de Mallorca, Spain

**Keywords:** Body fat, DXA, Cut-off, Qatar Biobank

## Abstract

**Background:**

Excessive body fat is the leading cause of many metabolic disorders. Therefore, assessing levels of body fat associated with risk of disease in specific populations is crucial. The present study aimed to identify optimal cut-off values of body fat composition including total body fat, body fat percentage, visceral fat, and trunk fat, in order to predict metabolic risk in the Qatari population.

**Methods:**

This cross-sectional study was based on Qatar Biobank data of 2407 Qatari adults (1269 male and 1138 female) aged 21–70 years old. Individuals’ height, weight and body fat percentage were obtained. Blood test data including lipid profile, blood glucose and HbA1c data were also obtained. The area under the curve was calculated using ROC analysis to obtain the body fat percentage associated with risk of disease.

**Results:**

The cut-off points for total fat for those aged < 40 were 34.0 kg, and for those aged ≥40 were 30.7 kg and 35.6 kg in men and women, respectively. The cut-off for body fat percent for those aged < 40 were 35.1 and 45.1%, and for those aged ≥40 were 34.8 and 46.3% in men and women, respectively. The cut-off points for trunk fat percent for those aged < 40 were 19.5 and 22.4%, and for those aged ≥40 were 21.6 and 23.4% in men and women, respectively. The cut-off points for visceral fat percent for those aged < 40 were 1.4 and 1.0%, and for those aged ≥40 were 1.9 and 1.4% in men and women, respectively.

**Conclusion:**

This study established Qatari adult-specific cut-off values of body fat for different age and gender groups.

## Background

The global prevalence of obesity nearly doubled between 1980 and 2008. The World Health Organization reported that 35% of adults worldwide aged over 20 years were overweight (34% men and 35% women) including 10% men and 14% women being considered as obese [1]. The prevalence of overweigh and obesity among Qatari population has reached an alarming rate of 70 and 41% respectively [2]. It is well known that the accumulation of excessive body fat may contribute to the development of cardiovascular risk factors, such as hypertension, insulin resistance, diabetes mellitus, and dyslipidaemia which contribute to cardiovascular diseases (CVD), such as coronary heart disease and stroke [3–5].

Accumulation of adipose tissue among obese individuals is an independent underlying cause of several chronic life-threatening diseases. It is therefore essential to assess body fat (BF) percentage.

Assessing BF and its distribution would be useful in screening for obesity and its metabolic risk factors [6]. It is well known that levels of body fat associated with metabolic risk vary between ethnic populations, age groups and genders [7]. For instance, results from the Chinese population reported that BF% was higher among people with low body mass index (BMI) compared to the European population [8]. Ethnic differences in levels of body fat and metabolic risks emphasise the need to establish population-specific cut-off values for body fat. Data investigating body-fat cut-off points associated with metabolic risk in Arabic countries are limited, with no data available in Qatar. Therefore, the overall aim of this study was to establish age and gender specific cut-off points for body fatness indicators, specific for the Qatari population.

## Methods

### Participants

The study sample was based on a subsample of the Qatar Biobank survey. Exclusion criteria of the study were non-Qataris, those aged below 21 or above 75 years old, pregnant women, and athletes. A random sample of 2802 was obtained from the Biobank survey data. Participants with incomplete measurements were excluded (*n* = 218). Participants with missing data about unpleasable body fat measurements or metabolic risk indicators were also excluded (*n* = 177). A net sample of 2407 Qatari participants (1269 males and 1138 females) was included in the analysis. Biobank recruitment and data collection protocols were approved by the Hamad Medical Corporation Ethics Committee. Institutional Review Board approval for this study was obtained from Qatar Biobank.

### Demographic and health data

Sociodemographic data were collected as part of a self-completed questionnaire. Data about educational level, age, gender, occupation and physical activity were collected. A trained nurse was available to assist participants completing the questionnaire upon request.

Data regarding health, family history and use of medication were collected by face-to-face interview, carried out by a trained nurse. Information about previous and current diseases experienced by the participants or their family members were collected. The interview also included data about the use of prescribed and over-the-counter medication.

Anthropometric measurements including weight, height, and waist and hip circumferences were recorded with participants wearing light clothes [9]. Measurements were taken by a trained registered nurse. For hip and waist circumference measurements, participants were asked to remove outer garments and the circumferences of hip and waist were measured by tape and recorded in centimetres.

### Cardio-metabolic data

Blood samples (approximately 60 ml) were collected by a registered nurse to measure participants’ total cholesterol levels (CHOL), high-density lipoprotein cholesterol (HDL-C), low-density lipoprotein triglycerides (TG), glycosylated glucose (HbA1c%), and fasting blood glucose. Blood pressure was measured using the Omron 705 automated device. Duplicate measurements were recorded for diastolic and systolic blood pressure measurements, with additional measurements taken if there was a 5 mmHg or more difference in the duplicate readings.

Metabolic syndrome (MS) was defined using the harmonised international definition [10]. In detail, high waist circumference (WC) was defined as WC ≥ 102 cm in men and ≥ 88 cm in women; low HDL as HDL < 1.04 mmol·L^− 1^ in men and < 1.29 mmol·L^− 1^ in women; high triglycerides as triglycerides ≥1.7 mmol·L^− 1^ or treatment with triglyceride-lowering drugs; high blood pressure as systolic blood pressure ≥ 130 mmHg or diastolic blood pressure ≤ 85 mmHg or treatment with pressure-lowering drugs; and high glucose as glucose ≥5.6 mmol·L^− 1^ or treatment with glucose-lowering drugs, and MS as 3 or more of the above conditions.

### Body fat data

Body composition was measured by full body dual energy X-ray absorptiometry (iDXA, General Electric) scan. Participants were asked to wear a gown and remove jewellery before undergoing the test, so any interference with the X-rays could be avoided. Moreover, participants were asked to lie flat on the scanning table and to stay still so the X-ray image could be taken through the scanning arm without getting blurred.

### Statistical analysis

To calculate the sensitivity, specificity, and area under the curve (AUC) for different adiposity measures in predicting MS, a user-written syntax *cutpt* in Stata was used. We used the Liu method to maximize the product of the sensitivity and specificity in the selection of the optimal cut point for predicting metabolic syndrome [11]. We calculated the above indicators in men and women. We also stratified the analyses by age groups. All analyses were conducted in Stata 15.1. *P* < 0.05 were considered as statistically significant.

## Results

Table 1 describes the study sample characteristics by gender. The mean age of the participants was 39.4 years. The mean BMI for women (29.5 kg/m^2^ ± 6.1) was higher than that for men (28.4 kg/m^2^ ± 5.0). The prevalence of metabolic syndrome was 18.0% in both genders. Women had higher total fat mass, total fat percent, and trunk fat percent compared to men (*P* < 0.001). Visceral fat mass and percent were higher in men than women (1.3 ± 0.8 kg vs 0.8 ± 0.5 kg and 1.4 ± 0.7% vs 1.0 ± 0.6% respectively) (*P*-value < 0.001).

**Table 1:** Sample characteristic by genders

	Total	Male	Female	***P***-value
	***N*** = 2407	***N*** = 1269	***N*** = 1138	
Age (years)	39.4 (11.2)	38.7 (10.6)	40.1 (11.7)	0.002
Education				< 0.001
Low	239 (9.9%)	96 (7.6%)	143 (12.6%)	
Medium	680 (28.3%)	388 (30.6%)	292 (25.7%)	
High	1486 (61.8%)	783 (61.8%)	703 (61.8%)	
BMI (kg/m2)	29.0 (5.6)	28.4 (5.0)	29.5 (6.1)	< 0.001
BMI categories				< 0.001
Normal	575 (23.9%)	308 (24.3%)	267 (23.5%)	
Overweight	900 (37.4%)	545 (42.9%)	355 (31.2%)	
Obese	932 (38.7%)	416 (32.8%)	516 (45.3%)	
Metabolism syndrome	434 (18.0%)	229 (18.0%)	205 (18.0%)	0.98
Total mass (kg)	79.2 (16.3)	84.4 (15.1)	73.5 (15.7)	< 0.001
Total fat (kg)	30.9 (10.7)	28.6 (10.3)	33.4 (10.6)	< 0.001
Total fat percent	38.6 (8.7)	33.4 (7.3)	44.5 (5.9)	< 0.001
Trunk fat (kg)	16.2 (6.4)	16.2 (6.6)	16.2 (6.2)	0.98
Trunk fat percent	20.0 (5.0)	18.8 (5.1)	21.4 (4.5)	< 0.001
Visceral fat (kg)	1.0 (0.7)	1.3 (0.8)	0.8 (0.5)	< 0.001
Visceral fat percent	1.2 (0.7)	1.4 (0.7)	1.0 (0.6)	< 0.001

Data are presented as mean and standard deviation (SD) for continuous measures, and n (%) for categorical measures. Significance was set at *P* < 0.05

The cut-off points for body fat are presented in Table 2. Total fat mass of 34.0 kg was associated with cardiometabolic risk among men and women under the age of 40 with AUC of 0.7 and 0.8 for men and women respectively. For individuals aged ≥40, total fat (AUC) of 30.6 kg (0.7) and 35.6 kg (0.6) was associated with cardiometabolic risk among men and women, respectively. The sensitivity of total fat, for those aged < 40 was higher among women (82.8%) compared to men (67.6%). Specificity of total fat for those aged < 40 was higher in men (79.0%) compared to women (69.5%). Men’s sensitivity and specificity for total fat for those aged ≥40 were higher in comparison to those for women in the same age range.

**Table 2:** Age-and-gender specific cutoff points for total fat associated with metabolic risk

Variable	Cut-point	Sensitivity	Specificity	AUC	Cut-point	Sensitivity	Specificity	AUC
	Men				Women			
Total fat, Age < 40	34.0	67.6	79.0	0.7	34.0	82.8	69.5	0.8
Total fat, Age ≥ 40	30.6	67.8	66.2	0.7	35.6	62.5	59.5	0.6
Percent fat, Age < 40	35.1	71.6	66.2	0.7	45.1	79.3	62.8	0.7
Percent fat, Age ≥ 40	34.8	70.6	60.1	0.7	46.3	57.6	57.1	0.6
Trunk fat, Age < 40	20.8	67.6	84.7	0.8	15.7	86.2	68.3	0.8
Trunk fat, Age ≥ 40	18.9	69.0	72.7	0.7	18.0	68.5	66.6	0.7
Trunk fat percentage, Age < 40	19.5	75.7	69.4	0.7	22.4	69.0	77.0	0.7
Trunk fat percentage, Age > =40	21.6	66.7	72.7	0.7	23.4	65.8	66.1	0.7
Visceral fat, Age < 40	1.2	78.1	73.1	0.8	0.8	85.7	81.9	0.9
Visceral fat, Age ≥ 40	2.0	64.8	83.8	0.7	1.0	75.7	67.0	0.7
Viseral fat percentage, Age < 40	1.4	71.2	74.1	0.7	1.0	85.7	81.2	0.8
Viseral fat percentage, Age > =40	1.9	72.9	64.9	0.7	1.4	66.9	74.8	0.7

Sensitivities and specificities are expressed in percentage (%), and cut-off values are expressed in kg. *AUC* Area under the curve expressed in percentage (%)

With regard to percentage body fat**,** the percentage body fat cut-off points (AUC) for men were 35.1% (0.7) and 34.8% (0.7) among those < 40 and ≥ 40 years, respectively. The corresponding figures for women were 45.1% (0.7) and 46.3% (0.6), respectively. Among men age < 40, percentage fat sensitivity was lower (71.7%) than for women (79.3%) in the same age range. However, among women age < 40, percentage fat specificity was less (62.8%) than that for men (66.2%) in same age range. The sensitivity and specificity of percentage fat for those aged ≥40 in women (57.6 and 57.1%) were much lower than those for men (70.6 and 60.1%) of the same age range, respectively.

The cut-off point (AUC) of trunk fat and trunk fat percent among men aged < 40 were 20.9 kg (0.8) and 19.5% (0.7) respectively. Corresponding values (AUC) for men aged ≥40 were 18.9 kg (0.7) and 21.6% (0.7) respectively. In women who were less than 40 years of age, the cut-off points (AUC) of truck fat (kg) and trunk fat % were 15.6 kg (0.8) and 22.4% (0.7). However, the corresponding values (AUC) for women with age of 40 and above were, 18.0 kg (0.7) and 23.4 kg (0.7).

The cut-off points (AUC) for visceral fat mass associated with metabolic risk among men and women with less than 40 years of age were 1.2 kg (0.8) and 0.8 kg (0.8) respectively. However, the corresponding values for men and women aged ≥40 were 2.0 kg (0.8) and 1.0 kg (0.7) respectively. Considering the visceral fat percent, the cut-off values (AUC) were as follows 1.4% (0.7) and 1.9% (0.7) for men aged < 40 years and men ages ≥40 years respectively; and 1.0% (0.8) and 1.4%(0.7) for women aged < 40 years and women ages ≥40 years respectively.

## Discussion

This study was conducted to develop gender and age specific cut-off points of BF% for healthy Qatari adults to determine their risk of having metabolic syndrome. These cut-off points of BF% have been created based on DEXA, anthropometric and biochemical data that have been collected from the study participants.

Body fat percentage is a better indicator of metabolic risk factors than body mass index [12]. The ability of BMI to diagnose obesity and risk of disease remains questionable [13]. The accuracy of BMI is limited due to the inability to measure body fat percentage directly from total body fat, total body lean mass, and total bone mass [14]. Different ethnic groups showed different results based on age and gender [15]. Therefore, additional cross-ethnic studies are required, as body fat percentage and BMI relations are affected by ethnicity, age and gender [16].

A study carried out on middle-aged Japanese men showed a link between BF% and CVD risks, such as diabetes mellitus, dyslipidaemia and hypertension [17]. Li et al. (2012) revealed that there is a relationship between BF%, type 2 diabetes, and metabolic syndrome. They used BF% from the Shanghai Diabetes Studies, which were 25% for men and 35% for women [14].

In our study, BF % cut-off values that were associated with metabolic syndrome for Qatari adults aged < 40 years old and ≥ 40 years old were 35.1 and 45.1% for men, and 34.8 and 46.3% for women. The result obtained from BF% showed a clearer link to metabolic syndrome risk for women, compared to men. This could be due to higher fat mass and lower lean body mass in women than men. Furthermore, women aged < 40 years had higher BF% area under the curves (AUC) for CVD risk factors than women age > 40 years. This high AUC of women < 40 years old means that the results are diagnostic for a large number of women in this age range, thus giving higher predictability for metabolic risk and allowing early intervention to be initiated.

One of many similar studies is a study conducted by Li et al. in 2017 aimed at determining cut-off values of BF% to estimate the risk of cardiovascular disease [14]. This was a cross-sectional study which included 3221 Chinese adults from two different ethnicities (2308 Han and 913 Mongolian). The study included 898 Han men and 355 Mongolian men, and 1410 Han women and 558 Mongolian women, with an age range of 20–80 years old. The BF% cut-off points were measured using bioelectrical impedance. In the Mongolian population, men had body fat cut-off points of 25% and women had cut-off points of 35%. The results were similar for the Han population [14]. Other studies in Korea supported the fact that BF% is a strong indicator for cardio-metabolic risk [18]. Kim et al. stated that BF% cut-off points in Korean populations are 21 and 37% for men and women, respectively [18]. Moreover, another study has established individualised BF% cut-off values for the Asian-Indian people, reporting that men have a cut-off point of 24.5%, while that of women is 38% [19].

The present study’s results of trunk fat indicated that the specificity of trunk fat to predict metabolic risk was higher in males aged < 40 (84.7%) than females aged < 40 (68.2%); whereas the sensitivity was higher for women (86.2) than in men (67.6) at age < 40 years.

One of the studies related to visceral fats is a study that involved 278 girls and 302 boys between the age of 3–19 years old [20]. Analysis of trunk fat was accomplished by DEXA. Specificity and sensitivity of trunk fat in boys were 92 and 87%, and in girls were 94 and 89% [20]. Researchers have stated that the difference between boys and girls might be attributed to differences in body construction (apple shape, gynoid shape), in addition to the specific skeletal structure of each gender.

Snijder and co-workers (2004) conducted a study to investigate the association between trunk fat and blood glucose levels. They revealed that there is a strong correlation between trunk fat and metabolic syndromes, such as diabetes [21]. Meanwhile, Taylor et al., (2012) have reported that abdominal adiposity increases the risk of diabetes and cardiovascular disease based on their study of the Indian population [22]. Trunk fat in this population was measured using DEXA that measured fat in the abdominal area, such as the liver, subcutaneous fat and other tissues as well [22].

Our results revealed the visceral fat cut-off points associated with metabolic risks were slightly higher in men in comparison to those of women in both age ranges, with 1.2 kg visceral fat cut-off value in men and 0.8 kg cut-off value for women under the age of 40. A result, 2.0 kg of visceral fat has been indicated as the borderline for men ≥40 to get a metabolic disease, while the borderline for women ≥40 is 1.0 kg of visceral fat.

One of the studies related to visceral fat is a cross-sectional study conducted by Amato et al., (2011) among Caucasian Sicilian subjects. The sample size was 1764 primary care (PC) patients, of whom 585 were men and 1179 were women, aged between 16 and 99 years old [23]. The researchers have indicated a strong relationship between the visceral adiposity index (VAI) and metabolic risk factors. In primary care patients < 30 years, 30–42 years, 42–52 years, 52–66 years, > 66 years, VAI cut off points were 2.52, 2.23, 1.92, 1.93, and 2, respectively [23].

Trunk and visceral fat percentages – unlike the trunk and visceral fat mass- may provide information about the visceral fat and trunk fat in relation to the total body mass. However, our analysis did not show a privilege of using percentage of fat over using mass of fat (specificity and sensitivity were comparable).

## Conclusion

This study established specific cut-off values of body fat parameters for Qatari adults of different ages and gender that can be used as a reference for determining obesity-related metabolic risks. The study has revealed that there is a strong relation between body fatness and the risk of metabolic diseases.

## Data Availability

The research is based on secondary data owned by Qatar Biobank. Authors do not have the authority to provide the data.
